# Splenic Infarct Masquerading as Myocardial Infarction

**DOI:** 10.7759/cureus.60138

**Published:** 2024-05-12

**Authors:** Kinza Moin, Maitha Al-Neyadi, Tarab Iqbal, Thiagarajan Jaiganesh

**Affiliations:** 1 Emergency Department, Tawam Hospital, Al Ain, ARE

**Keywords:** abdominal pain, thromboembolism, diabetes, myocardial infarction, splenic infarct

## Abstract

Splenic infarction is an uncommon cause of abdominal pain. Diabetes increases the risk of blood vessel occlusion and consequent tissue infarction due to blood vessel abnormalities such as atherosclerosis or thrombosis. Systemic thromboembolism secondary to myocardial infarction is associated with an increased risk of morbidity and mortality. We report the case of a 45-year-old woman with uncontrolled diabetes who presented to the emergency department with the sole complaint of left upper quadrant pain. Upon investigations, it was discovered that she had concomitant splenic and myocardial infarctions. This case demonstrates the significance of thrombotic complications in various organs in patients with uncontrolled diabetes mellitus. Clinicians should have a high suspicion of acute vascular infarction of several organs in poorly controlled diabetic patients with nonspecific symptoms.

## Introduction

Splenic infarction is not typically a physician's top differential diagnosis when a patient arrives at the emergency department complaining of abdominal pain. It occurs due to compromised arterial blood flow to the spleen, particularly in association with thromboembolic, vascular, and hematologic conditions [1]. Diabetes also induces a thrombotic state through endothelial dysfunction and platelet hyperreactivity due to underlying oxidative stress and inflammation [2]. Systemic thromboembolism associated with cardiovascular conditions can often cause splenic infarction [3]. Here, we report a case of concomitant myocardial and splenic infarction in a middle-aged woman with the chief complaint of left upper quadrant pain.

## Case presentation

A 45-year-old female presented to the emergency department with left upper quadrant pain, which extended to her back. The pain was dull, moderate in severity, and accompanied by nausea and repeated episodes of vomiting. She had a four-month history of type 2 diabetes with poor compliance to oral hypoglycemic medications with a baseline hemoglobin A1C (HbA1c) of 14%. She was seen in the ambulatory health clinic earlier in the day and was referred to our tertiary care hospital for symptom control and further management. 

On arrival to the emergency department, the patient was alert and oriented. Vital signs showed a blood pressure of 114/98 mmHg, a heart rate of 98 beats per minute, a respiratory rate of 18 breaths per minute, and an oxygen saturation of 98% on room air. Random blood glucose was 23 mmol/L (4-6 mmol/L) with normal blood ketones. She was given a stat dose of insulin regular 10 units subcutaneously for hyperglycemia. 

Upon clinical examination, the patient looked in mild to moderate distress. She had a tense abdomen and tenderness in the mid-epigastric and left upper quadrant region with no rebound tenderness or guarding. Based on the symptoms and physical findings, complete blood count, urea and electrolytes, liver function tests, lipase, and abdominal X-ray were ordered by the emergency department physician. 

**Figure 1:**
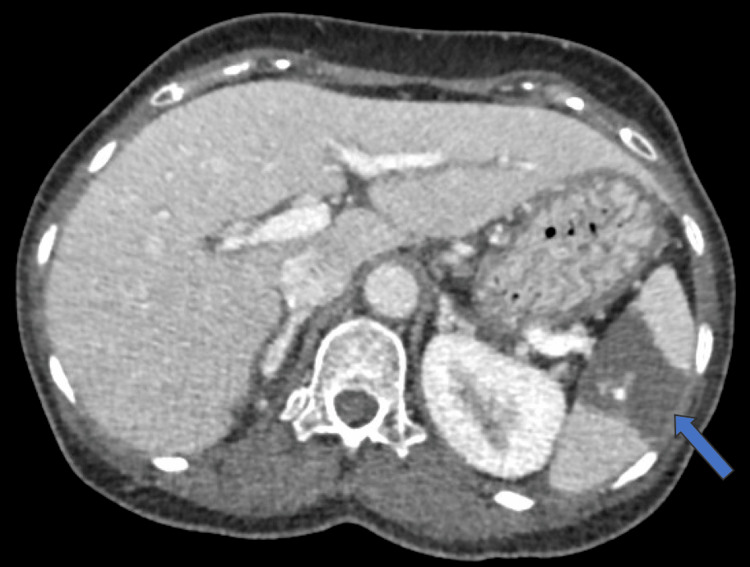
Axial contrast-enhanced abdominopelvic tomography scan showing a large hypodense area in the spleen (splenic infarction; blue arrow)

**Figure 2:**
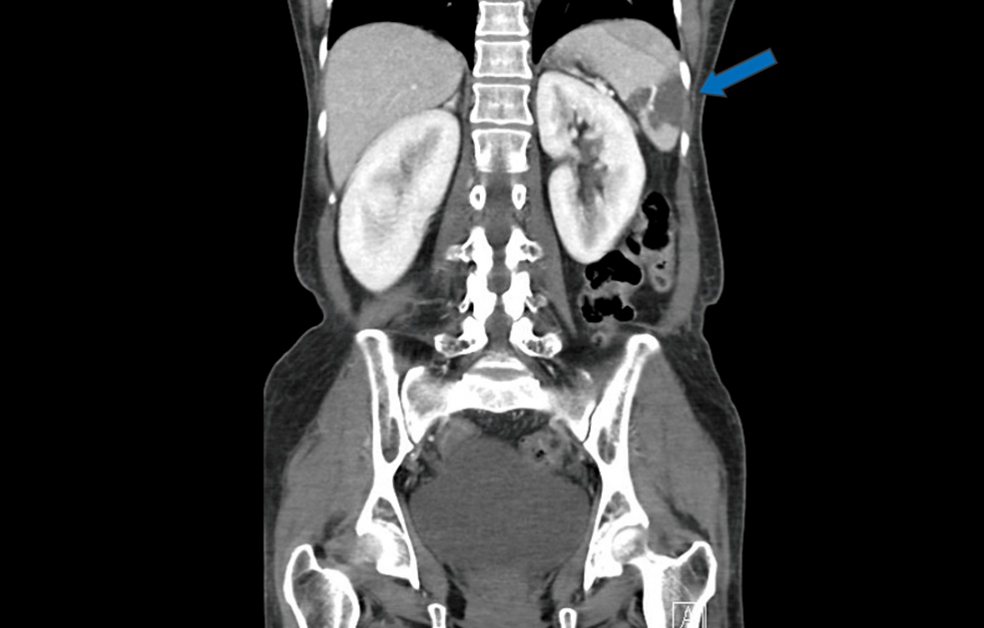
Coronal contrast-enhanced abdominopelvic tomography scan showing a hypodense area in the spleen (splenic infarction; blue arrow)

A bedside point-of-care venous blood gas (VBG) showed a pH of 7.30, base excess -4.8, bicarbonate 20 mmol/L, and normal lactate. Laboratory results showed a total leucocyte count of 15.6 × 10^9^/L (4.0-11.0 × 10^9^/L), with a differential leucocyte count of 12% neutrophils, 2% lymphocytes, and 1% eosinophils. The patient had normal renal function, electrolytes, and liver function tests. Abdominal X-ray showed no air-fluid levels on upright film, no bowel dilatation, and nonspecific bowel gas distribution. The patient was treated with IV crystalloids, anti-emetics, and analgesia. Despite strong analgesia and symptomatic management, her pain did not improve. Due to the strong increment toward the surgical cause of abdominal pain, computed tomography (CT) of the abdomen and pelvis with contrast was ordered which showed a large non-enhancing area in the spleen suggestive of splenic infarct (Figure 1 and Figure 2). Multidisciplinary teams including general surgery, hematology, and internal medicine were consulted. General surgery examined the patient and concluded that there was no indication for urgent surgical intervention.

**Figure 3:**
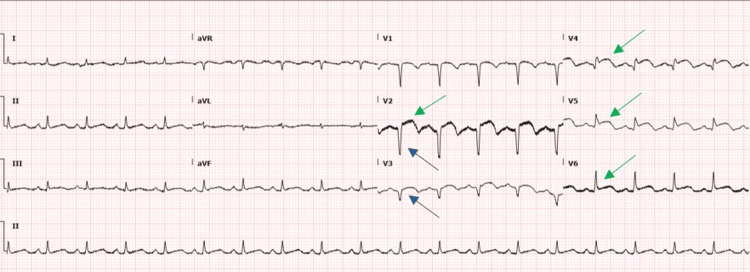
ECG showing STE in leads V2, V4, V5, and V6 (green arrows) and Q waves in V2 and V3 (blue arrows) ECG: electrocardiogram; STE: ST-segment elevation

Despite 20 ml/kg of IV crystalloid bolus and repeated administration of strong opioid analgesia, the patient's pain did not significantly improve. Repeated VBG showed worsening of metabolic acidosis with a pH of 7.2, bicarbonate 18 mmol/L, base excess -5, and normal lactate. The patient was planned for admission, and during her routine investigations, an electrocardiogram (ECG) was done for her which showed a heart rate of 113, ST elevations in leads V2, V4, V5, and V6, and Q waves in V2 and V3 suggestive of sub-acute antero-septal myocardial infarction (Figure 3). Her first set of troponins was 150 ng/L (<14.0 ng/L). The patient was given aspirin 300 mg, ticagrelor 180 mg, and a therapeutic dose of enoxaparin and later was admitted to the coronary care unit. On admission day 1, a coronary angiogram was done which showed 100% occlusion in the left anterior descending coronary artery after the first diagonal (D1) branch with a blunt stump. The echocardiogram showed a left ventricle (LV) ejection fraction of 35%, moderate-severe LV dysfunction, and regional wall motion abnormalities in the apex, anterior septum, apical lateral, and anterior wall segment akinesia. No clot or intramural thrombus was seen; however, echocardiogram with contrast was not done for this patient. Based on her echocardiogram and coronary angiogram findings, it was decided to medically manage her with dual antiplatelets and therapeutic doses of enoxaparin. Her blood glucose levels were managed with sliding scale insulin regular and insulin glargine 12 units subcutaneously. 

Investigations for an underlying thrombophilia with factor V Leiden screen, antithrombin III, protein C, and protein S along with immunology workup including antinuclear antibody (ANA) titers, extractable nuclear antigen antibodies (ENA screen), and beta-2 glycoprotein immunoglobulin G (IgG) and immunoglobulin M (IgM) were carried out, which all came negative. She was managed medically for both splenic and myocardial infarction.

The patient had an uneventful five-day course in the hospital and showed good clinical recovery, repeated CT scan of the abdomen with contrast showed no progression of the splenic infarction, and an ECG done before the discharge showed partial resolution of ST changes. She was later discharged on aspirin 100 mg, metformin 500 mg, glimepiride 1 mg, atorvastatin 80 mg, and edoxaban 30 mg with close follow-up in the cardiology and hematology clinic.

## Discussion

Splenic infarction as a cause of abdominal pain is one of the rarest presentations in literature; nevertheless, it remains a peculiar and rising presentation in clinical practice [1]. As documented in the literature, splenic infarction is an uncommon and underdiagnosed condition worldwide, accounting for only 0.016% of admissions over a 10-year period as reported in a single academic center study [4]. A splenic infarction results due to tissue necrosis from parenchymal ischemia, which happens when the arterial blood supply through the splenic artery is cut off. In almost half of the cases, patients present with left-sided abdominal pain, described as dull and non-radiating. Roughly three-quarters present with nonspecific and poorly localized abdominal pain, whereas less than one-third present with no abdominal symptoms. Nausea or vomiting can occur with abdominal pain in nearly 30% of the patients. According to the data, around 30-36% of patients may develop a fever with a tympanic temperature above 38°C [1].

Patients with heme-oncologic conditions, especially malignancies, sickle cell anemia, myelofibrosis, and those with other known hypercoagulable disorders are at a greater risk of splenic infarction [5].

In about 67% of the cases, a thromboembolic phenomenon was noted to be the main cause of splenic infarcts, mainly due to embolized atherosclerotic aortic debris, thrombotic components from the LV, particularly in patients with dilated cardiomyopathy, myocardial infarction, and infected heart valve vegetations [3]. The rate of thromboembolism post-myocardial infarction secondary to LV thrombus varies between 3% in patients on therapeutic anticoagulation and 19% in those with poor compliance to anticoagulant medications [6]. Although rare, thromboembolic complications have been reported in about 2% of patients following an acute myocardial infarction [7]. Virchow's triad which includes blood stasis due to regional wall abnormalities, hypercoagulability, and endocardial injury secondary to ischemia and inflammatory response predisposes to thrombus formation after myocardial infarction. LV thrombus can develop within 24 hours following acute myocardial infarction [6]. Diabetes leads to a prothrombotic state by affecting coagulation processes, endothelial function, and platelet activity. Dysregulation of signaling pathways leads to increased platelet aggregation by enhanced adhesion, activation, and aggregation. Multiple factors in diabetes, such as hyperglycemia, insulin resistance, inflammation, and oxidative stress, contribute to these changes. Accelerated atherosclerosis in diabetic patients is responsible for macrovascular and microvascular complications. Patients with type 2 diabetes are at a two- to fourfold increased risk of coronary artery disease and stroke as compared to the general population [2]. Uncontrolled diabetes was the only significant predisposing risk factor in our patient.

The spleen's function in myocardial infarction hasn’t traditionally attracted a great deal of research. A recent study on rats and pigs revealed that the spleen is crucial for cardio-protection, which works by remote ischemic conditioning via efferent vagal nerves [8]. There may be parallels that can be derived between the heart and the spleen, in the sense that both receive a nearly similar percentage of cardiac output [9]. Despite this, concurrent myocardial and splenic infarction remains to have limited literature documented on it.

Concurrent cardiac, splenic, and renal infarcts have been documented in a patient with COVID-19 owing to the hypercoagulable state associated with the virus [10]. Another case report described a potential thromboembolic event causing splenic infarction and myocardial injury in a patient with underlying atrial fibrillation and diabetes as the risk factors [11]. To the best of our knowledge, this is the first reported case of concomitant splenic and myocardial infarction in a patient without hypercoagulable disorder, atrial fibrillation, or a positive COVID-19 result. Our patient had diabetes as the sole important risk factor with no prior history of cardiac valve disease or atrial fibrillation and a negative thrombophilia profile; however, it could be postulated that along with diabetes-induced thrombosis, cardioembolic phenomenon due to LV mural thrombus formation after acute anterior myocardial infarction could have been the cause of a splenic infarct in this patient.

No specific laboratory tests can diagnose splenic infarction; however, two-thirds of the patients can have neutrophilia with a left shift. Due to tissue injury secondary to infarction or infection, lactate dehydrogenase (LDH) can be high in the majority of patients. D-dimer could be raised secondary to its established link between thromboembolic events and clotting disorders with fibrin degradation. CT with contrast remains the definite and superior mode of investigation. Gadolinium-enhanced magnetic resonance imaging (MRI) is also another reliable diagnostic option. According to one study, ultrasound (US) has a reported diagnostic value of up to 18% and is only useful if spleen parenchyma can be identified. In cases of large infarctions, color Doppler US can reveal areas with no blood flow. Second-generation US has high specificity in diagnosing splenic infarction reaching up to 100% [12]. We observed left-shift neutrophilia in our patient, with negative results for LDH and D-dimer.

Treating uncomplicated splenic infarction is based on the etiology, and those with an unidentified cause require admission for monitoring and evaluation. Fortunately, uncomplicated cases requiring no intervention recover uneventfully in 7-14 days. Noninfective cases require supportive care with hydration and symptom control for pain, nausea, and vomiting. In patients with septic emboli, antibiotics should be added to the therapy, and extensive cardiac evaluation is warranted. Patients with hematological or immunological backgrounds should obtain subspecialty consultations to treat the patient's predisposing risk factors [13]. During her stay, our patient received medical treatment for splenic and myocardial infarction, as well as aggressive management of her risk factors.

Infarction of the spleen can cause superimposed infection and may result in abscess formation. Infarcts can also develop into hemorrhagic transformation necessitating splenectomy. Pseudocyst formation, splenic rupture, and aneurysm are other well-known complications [13].

## Conclusions

Physicians should keep splenic infarction as a differential diagnosis in patients with abdominal pain. It is essential to identify the underlying causes of splenic infarction to avoid complications and recurrences. The decision to prescribe anticoagulants should be based on the cause, patient's comorbidities, assessment of risk and benefits, and probability of recurrences. Diabetic patients with nonspecific complaints should raise clinicians' suspicion of acute vascular infarctions in multiple organs. Atypical signs of myocardial events such as epigastric pain, nausea, and vomiting should not be overlooked in the emergency department.
